# Corrigendum: ‘Nanopore-only assemblies for genomic surveillance of the global priority drug-resistant pathogen, *Klebsiella pneumoniae*’

**DOI:** 10.1099/mgen.0.001084

**Published:** 2023-08-09

**Authors:** Ebenezer Foster-Nyarko, Hugh Cottingham, Ryan R. Wick, Louise M. Judd, Margaret M. C. Lam, Kelly L. Wyres, Thomas D. Stanton, Kara K. Tsang, Sophia David, David M. Aanensen, Sylvain Brisse, Kathryn E. Holt

**Affiliations:** ^1^​ Department of Infection Biology, London School of Hygiene and Tropical Medicine, London, UK; ^2^​ Department of Infectious Diseases, Central Clinical School, Monash University, Melbourne, VIC, 3004, Australia; ^3^​ Centre for Genomic Pathogen Surveillance, Big Data Institute, Li Ka Shing Centre for Health Information and Discovery, Nuffield Department of Medicine, Oxford University, Oxford OX3 7LF, UK; ^4^​ Institut Pasteur, Université Paris Cité, Biodiversity and Epidemiology of Bacterial Pathogens, Paris, France

## Full-Text

In the published version of the article, there were errors in the read accessions provided in Table S1, which are now fixed. The updated file (TableS1_v2_13March2023.csv) can be accessed on Figshare using the DOI: 10.6084 /m9.figshare.19745608. Furthermore, we would like to correct the following sentence in the Data Summary section of the published version: ‘Illumina reads have been deposited in the NCBI SRA, under the BioProject ID: PRJEB6891, and ONT reads (SUP basecalled) have been deposited under BioProject PRJNA646837.’ The sentence should read: ‘Illumina and ONT reads have been deposited in the NCBI SRA, under the BioProject IDs: PRJEB6891, PRJNA646837 and PRJNA351909.’

The authors apologise for any inconvenience caused by these errors.

