# Supplementary material for: Nanopore-only assemblies for genomic surveillance of the global priority drug-resistant pathogen, Klebsiella pneumoniae
Source: Microb Genom. 2023 Feb 8;9(2):mgen000936. doi: 10.1099/mgen.0.000936 (PMC9997738; doi:10.1099/mgen.0.000936)
Supplement: Supplementary material 1 [file mgen-9-936-s001.pdf]

**Nanopore-only assemblies for genomic surveillance of the global priority drug-resistant pathogen, *Klebsiella pneumoniae***

Ebenezer Foster-Nyarko, Hugh Cottingham, Ryan R. Wick, Louise M. Judd, Margaret M. C. Lam, Kelly L. Wyres, Thomas D. Stanton, Kara K. Tsang, Sophia David, David M. Aanensen, Sylvain Brisse, Kathryn E. Holt

## SUPPLEMENTARY FIGURES

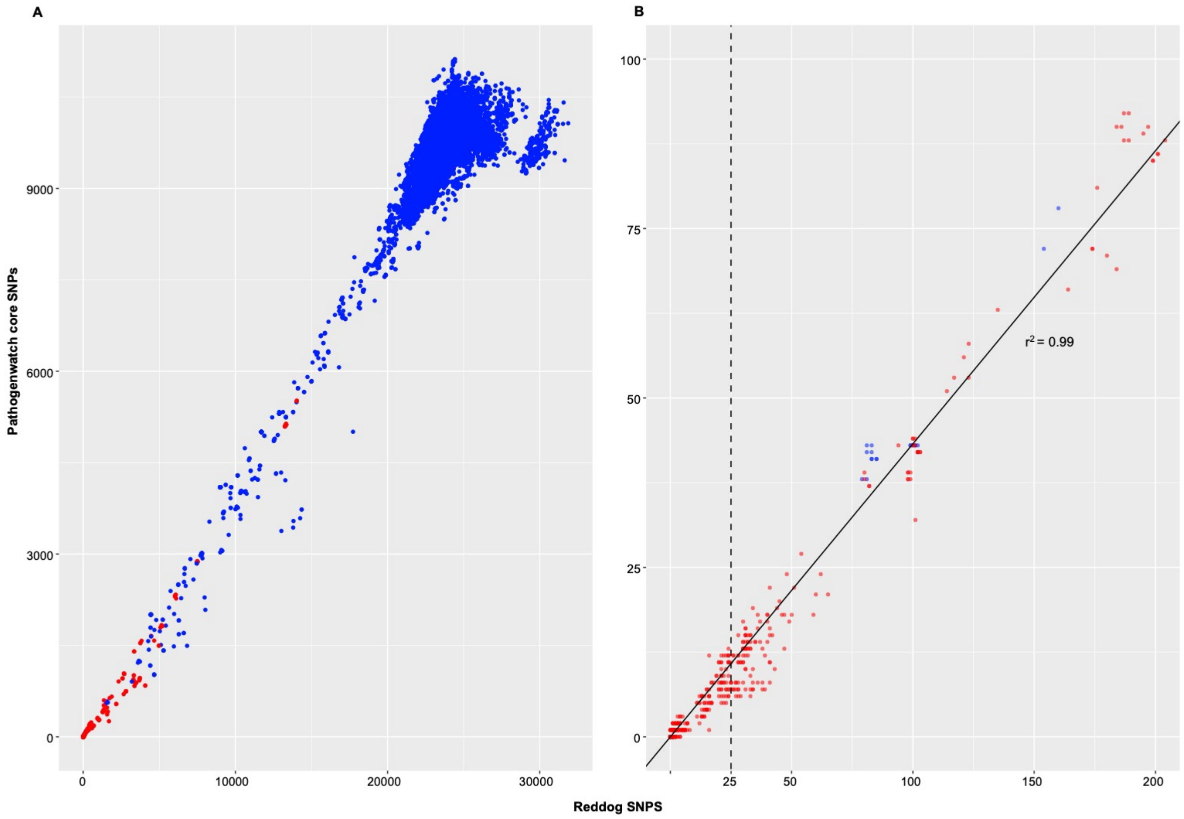


**Supplementary Figure 1. Comparison of Pathogenwatch distances with genome-wide mapping-based SNP distances, across n=270 diverse *Klebsiella pneumoniae* clinical isolates using Illumina data.** Y-axis shows Pathogenwatch pairwise distances (based on the core gene set of 1,972 genes within the Pathogenwatch *Klebsiella pneumoniae* core genome scheme) using Illumina assemblies; x-axis shows genome-wide mapping-based pairwise SNP distances (based on all variant sites for which alleles were called in ≥95% genomes, when

mapping Illumina reads to the NTUH-K2044 reference genome as described in reference 40). Data points represent pairwise distances, coloured to indicate pairs of genomes with the same (red) or different (blue) 7-locus sequence type. (**A**) All pairwise distances. (**B**) Zoom in to data points with pairwise genome-wide SNP distances ≤200; linear regression line is shown (solid line), adjusted $R^2$=0.9914 indicating very good model fit, slope=0.432; dashed line shows x=25 genome-wide SNPs, which is a common threshold used to identify putative transmission clusters. According to the linear regression, this is equivalent to a Pathogenwatch distance of 10.8 SNPs (i.e., threshold n=10).

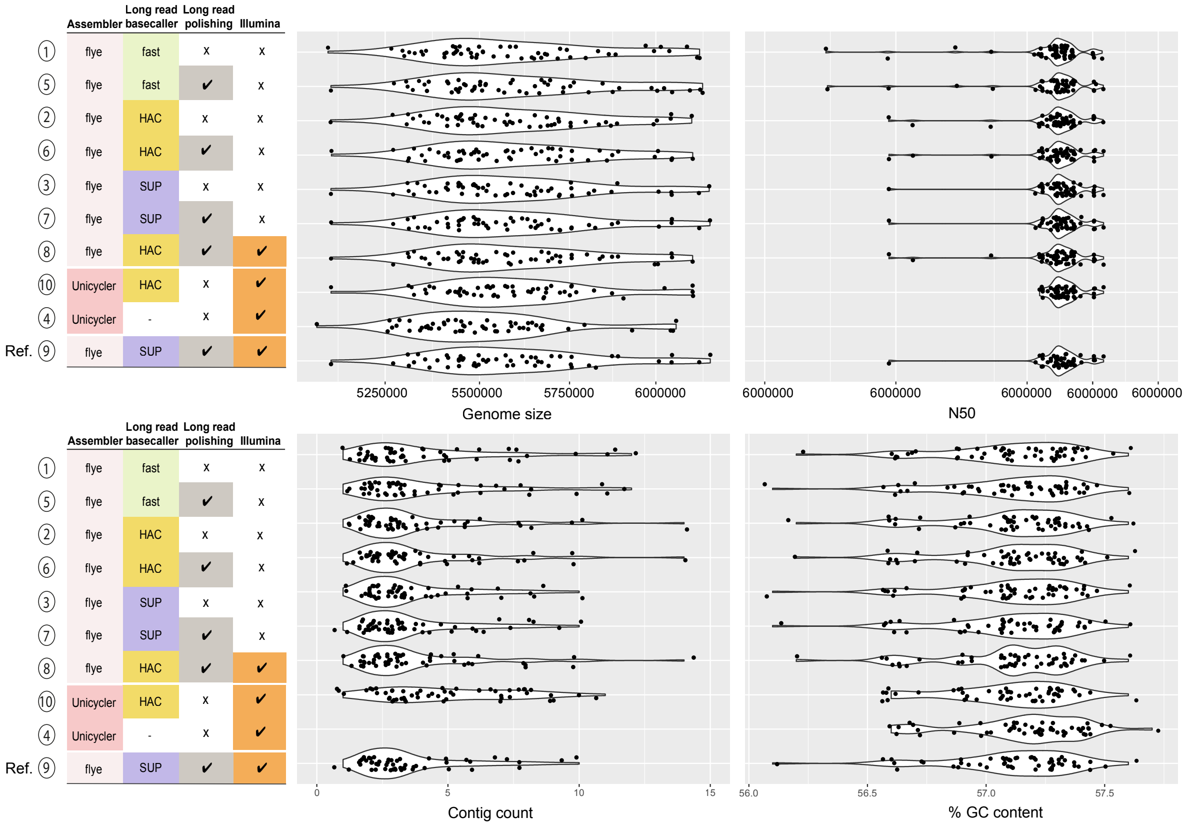


**Supplementary Figure 2. Distributions of sequencing metrics stratified by assembly type.** Assembly types are numbered 1 to 10 as in **Figure 1**. **(A)** Genome size. (**B**) N50. N50 distribution for Illumina is not shown as it generates draft assemblies with N50 < 1 Mbp (range, 69194 – 612419; median, 274529) (**C**) Contig count. Similar to panel B, contig count distribution for Illumina is not shown as it generates draft assemblies with >20 contigs (range 32 – 315; median, 92) (**D**) %GC content. The y-axis shows the various assemblies under comparison, with the gold standard hybrid ONT / Illumina reference assembly

(SUP+Medaka+pilon); while the x-axis displays the genome size, N50, contig count and %GC content distribution, respectively.

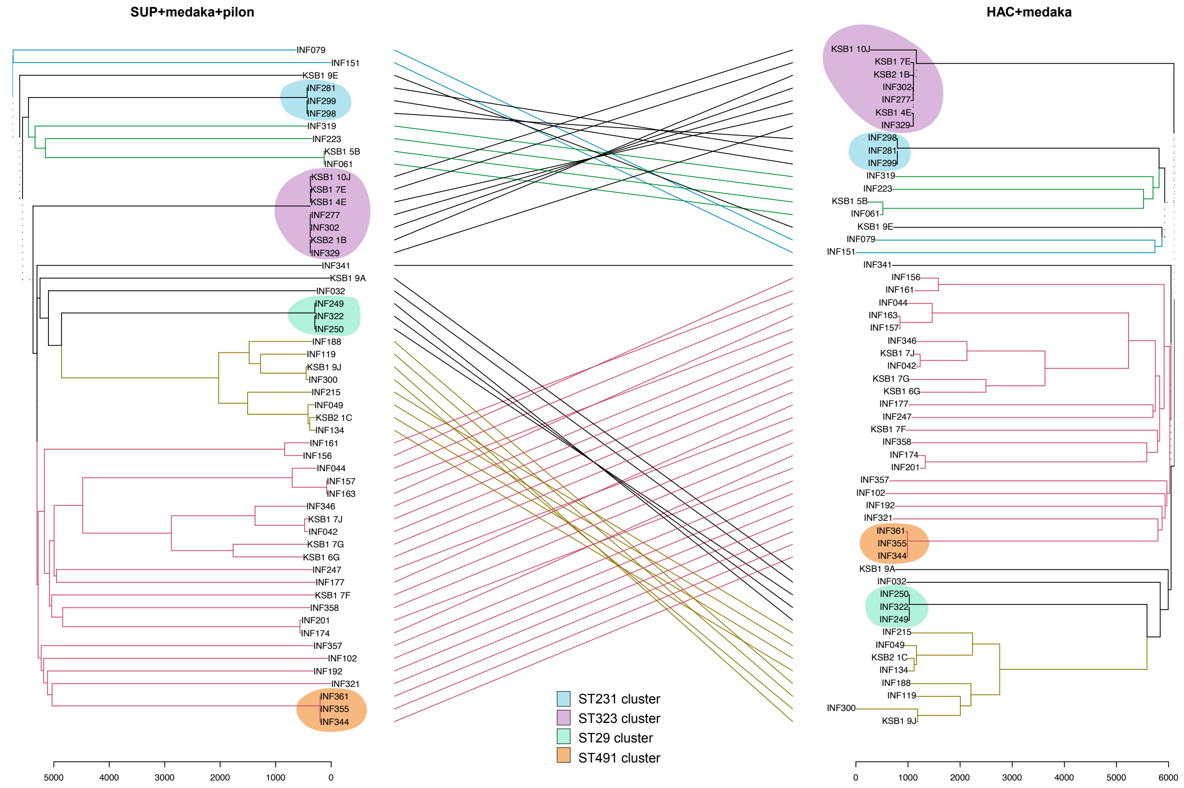


**Supplementary Figure 3.** A tanglegram depicting the alignment between a Neighbor-Joining tree produced from HAC+medaka assemblies and the ONT / Illumina hybrid reference tree. The tree yields an entanglement coefficient of 0.35 and a Robinson-Foulds distance of 22, indicating a good alignment. However, several clades are discordant with the reference tree, represented by the dotted lines. Coloured lines join matching tip labels for common subtrees, while black lines indicate subtrees that are not common between the two trees. Errors in the ONT-only assembly result in discordant branch lengths between the two trees.

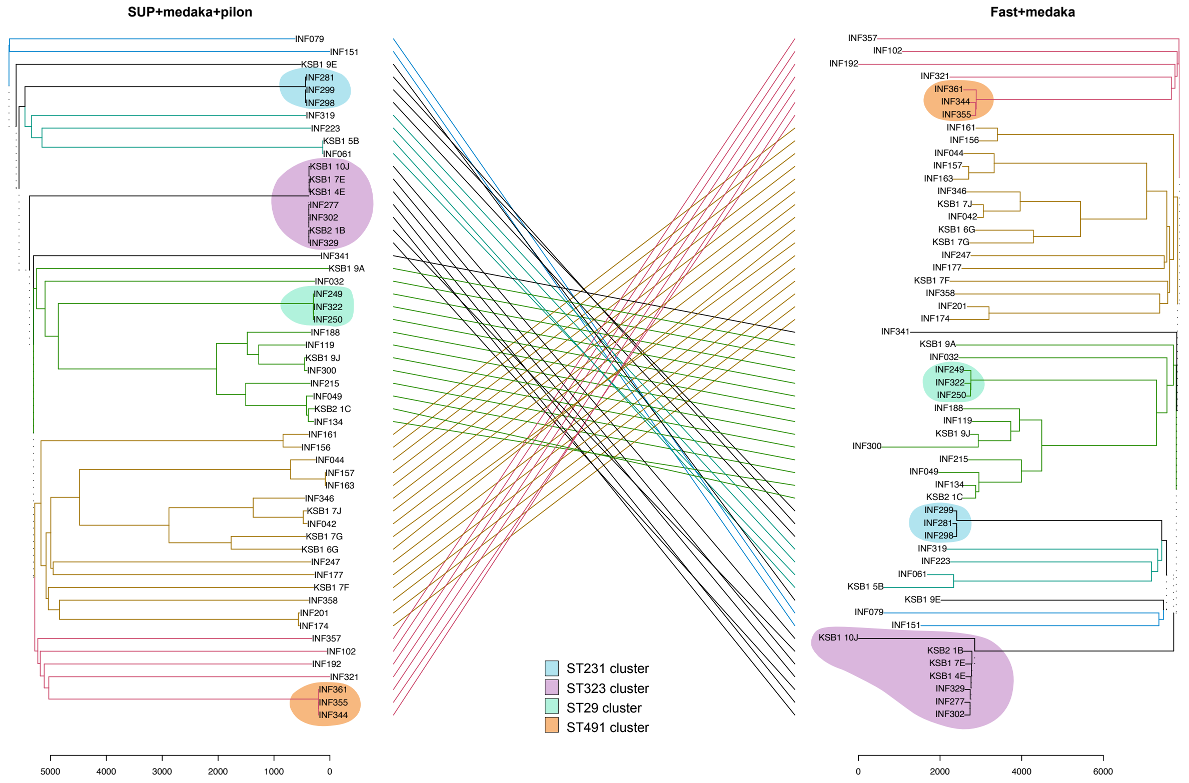


**Supplementary Figure 4.** A tanglegram depicting the alignment between a Neighbor-Joining tree produced from Fast+medaka assemblies and the ONT / Illumina hybrid reference tree. The tree yields an entanglement coefficient of 0.96 and a Robinson-Foulds distance of 102, indicating a very poor alignment. Coloured lines join matching tip labels for common subtrees, while black lines indicate subtrees that are not common between the two trees. Dotted lines represent distinct subtree present on either tree. Errors in the ONT-only assembly result in discordant branch lengths between the two trees.
